# Long Non-Coding RNA Cancer Susceptibility Candidate 9 Regulates the Malignant Biological Behavior of Nasopharyngeal Carcinoma Cells by Targeting miR-497-5p/Wnt3a/β-catenin Signaling Pathway

**DOI:** 10.3389/fonc.2022.807052

**Published:** 2022-03-28

**Authors:** Yue Lei, Wenlong Luo, Qiuyue Gong, Lan Luo, Wuyang Jing

**Affiliations:** ^1^ Department of Otorhinolaryngology Head and Neck Surgery, The Second Affiliated Hospital of Chongqing Medical University, Chongqing, China; ^2^ Laboratory Research Center, The First Affiliated Hospital of Chongqing Medical University, Chongqing, China

**Keywords:** lncRNA, nasopharyngeal carcinoma, CASC9, miR-497-5p, Wnt3a

## Abstract

Nasopharyngeal carcinoma (NPC) is a major kind of head and neck epithelial carcinoma. Increasing evidences reveal that long noncoding RNAs are considered as vital regulators in tumorigenesis and progression. Although previous studies have found that cancer susceptibility candidate 9 (CASC9) highly expresses in NPC, the underlying mechanisms need to be further studied. In this study, we found that CASC9 was overexpressed and associated with worse prognosis in NPC. CASC9 knockdown significantly inhibited the cell proliferation, migration and invasion *in vitro* and enhanced the sensitivity of tumor cells to cisplatin and paclitaxel. Mechanism research confirmed CASC9 regulated the malignant biological behavior of nasopharyngeal carcinoma cells by targeting miR-497-5p/Wnt3a/β-catenin signaling pathway. The present study might provide a novel mechanism for tumorigenesis and progression of NPC and contribute to the development of an effective molecular target therapy.

## Introduction

As a major kind of head and neck epithelial carcinoma, nasopharyngeal carcinoma which is characterized by remarkable racial and geographic tendency presents a relatively high incidence rate in China and Southeast Asia (1–3). Despite the application of chemoradiotherapy have significantly improved the survival, diagnostic delay does not seem to improve and some patients still rapidly progress to locally advanced or metastatic diseases (4, 5). Therefore, further exploration of the underlying mechanisms in NPC is essential and quite urgent for the improvement of early diagnosis and effective treatment.

Long noncoding RNAs (LncRNAs) were considered as vital regulators in the tumorigenesis and progression of a variety of malignancies, including NPC (6–8). Previous studies have discovered that a few dysregulated lncRNAs are related to NPC progression *via* regulating cell proliferation, apoptosis, migration, invasion and drug resistance, such as AFAP1-AS, LET, LINC00958 and HOXC13-AS (9–11). LncRNA CASC9 was firstly reported in esophageal squamous cell carcinoma as an oncogene in 2015, and was subsequently confirmed to be associated with tumorigenesis and progression of malignant tumors including gastric cancer, hepatocellular carcinoma and breast cancer (12–14). Recently, Su et al. have found that CASC9 highly expressed in NPC and was correlated with poor prognosis (15). However, CASC9-related researches on NPC is still very limited and its functions and underlying mechanisms remain poorly understood.

In this study, we further confirm that CASC9 is significantly up-regulated in NPC and is closely correlated with poor prognosis. Moreover, we firstly discovered that CASC9 activates Wnt3a/β-catenin signaling pathway through miR-497-5p, which promoted NPC cells proliferation, migration and invasion. Collectively, our results identified the prominent functions of the CASC9/miR-497-5p/Wnt3a axis in NPC, which may provide a novel insight for the treatment of NPC.

## Materials and Methods

### Bioinformatical Analysis

The analysis of gene differential expression was carried out by using the R/Bioconductor. LncRNAs for which |log2FC| was >3 and adj.P.Value was <0.001 were considered to be differentially expressed. We used the Gene Expression Profiling Interactive Analysis (GEPIA, http://gepia.cancer-pku.cn/) database to generate survival curves and identify their correlation to gene expression. The potential binding sites of miR-497-5p and CASC9 were predicted by bioinformatics analysis using StarBase online (https://starbase.sysu.edu.cn/).

### Clinical Samples and Cell Lines

Thirty-two pairs of NPC tissues and adjacent non-tumor tissues were collected by the Second Affiliated Hospital of Chongqing Medical University, during September 2016 to June 2019. Tissues were stored in RNAsafeguard liquid (Manassas, VA, USA) at -80°C and fixed by formalin and embedded by paraffin. According to current clinical guidelines for the treatment of nasopharyngeal carcinoma, intensity-modulated radiotherapy (IMRT) is the routine treatment for NPC in stage I (16, 17). All patients with stage II~IV NPC received radiotherapy with concurrent cisplatin-based chemotherapy. Detailed information about the clinicopathological characteristics of all patients was collected and shown in **Supplementary Table 1**. All cases were followed up in our inpatient and outpatient hospital departments. Follow-up ranged from the end of treatment to September, 2021. All the patients were followed up once every 3 months during the first year, followed by one visit every 6 months during the second to fifth year. All patients had voluntarily signed a written consent before samples were collected. And this study was approved by the Ethics Committee of the Second Affiliated Hospital of Chongqing Medical University.

NPC cell lines 5-8F, HONE-1, CNE-2, HNE-1 and nasopharyngeal epithelial cell line NP69 were obtained from the American Type Culture Collection (Biomars, Beijing, CHN). All cell lines were cultured in RPMI-1640 (Gibco, Life Technologies, Carlsbad, CA) supplemented with 10% fetal bovine serum (FBS, Gibco) at 37°C.

### RNA Extraction and qRT-PCR

Total RNA from cells or tissues was extracted using Trizol reagent (Takara, Japan) according to instructions. The qRT-PCR, based on SYBR Premix Ex Taq (Takara, Japan) on Illumina Eco Real-Time PCR System and Bio-Rad CFX Connect Real-Time PCR Detection System, was used to detect the expression of related gene in clinical specimens and cell lines. Primers used in this study were listed in **Supplementary Table 2**.

### Western Blot Analysis

The cells were lysed in a RIPA lysis buffer (Thermo Fisher, USA) and supplemented with a protease inhibitor on ice for 30 min, followed by centrifugation for 15 min to collect the supernatant. Western blotting was performed as previously reported (18).

### MTT Assay

Cell proliferation was measured with MTT assay. Cells were seeded in 96-well plates at a density of 1 × 10^3^ cells/well. The absorbance was tested at 570 nm wavelength with a microplate reader.

Cisplatin and paclitaxel with concentration gradient were added to the media to explore the relationship between CASC9 expression and chemosensitivity in NPC. MTT was performed and analyzed as described above.

### Colony Formation Assay

Cells were seeded in 6-well plates at a density of 500 cells/well. After incubation for 2 weeks, cells were fixed with 4% paraformaldehyde and stained with crystal violet. Colonies were counted using microscopy.

### Flow Cytometry

In order to analyze cell cycle distribution by flow cytometry, cells were collected and fixed overnight at 4°C in 70% ethanol. The results were analyzed with Modfit LT software (Verity Software House, USA).

### Cell Migration and Invasion Assay

The horizontal migratory abilities was determined using wound healing. We paved cells within six-well plates (4×10^5^ per well). After 24 hours, the cells that migrated to the empty space were observed. Transwell assay were used to determine cell vertical migration and invasion potential. About 2×10^4^ cells were seeded into the upper chamber with an 8 μm porous membrane, while 600μl of the medium supplemented with 20% FBS was placed into the lower chamber. After culturing for 24h, the migrated cells on the lower surfaces were fixed and stained with 0.5% crystal violet. For the invasion assay, the membranes of the transwell chambers were precoated with Matrigel (Sigma, USA).

### Fluorescence *In Situ* Hybridization

Cy3-labeled probe sequences for CASC9 was constructed by Genepharma (Shanghai, China). RNA FISH were performed for analysis of the localization of CASC9 in cell lines using fluorescent *in situ* hybridization kit (Genepharma, Shanghai, China).

### Dual Luciferase Reporter Gene Assay

The interactions within CASC9, miR-497-5p and Wnt3a were determine luciferase reporter assay. Vectors were constructed using pSI-Check2-based plasmid (Promega, Madison, USA) containing CASC9 or Wnt3a mRNA 3’-UTR. 293T and HONE-1 cells were co-transfected with the plasmids and miR-497-5p mimics (GenePharma, Shanghai, China). After 48h transfection, the luciferase activity was measured.

### *In Vivo* Xenograft Model

Four-week-old BALB/c nude mice were obtained from the Experimental Animals Center of Chongqing Medical University. Lentivirus mediated shRNA interference targeting (shCASC9) and an empty lentiviral vector (sh-NC) were constructed by GenePharma (Shanghai, China). After transfection, approximately 5×10^6^HONE-1 cells were resuspended in 100 μl PBS and subcutaneously injected into the back of nude mice. The tumor volume was measured every 7 days until the end of the observation according to the formula: V = length × width^2^/2. All the mice were sacrificed after injection for 35 days and the tumor specimens were isolated for further analysis.

### Statistical Analysis

All statistical tests were performed on SPSS 22.0. Measurement data are repeated more than three times and presented as mean ± standard deviation (SD). Statistical analyses were performed with Student’s t-test. Kaplan-Meier analysis was used to evaluate the cumulative survival probability. P < 0.05 was considered as statistically significant.

## Results

### CASC9 Is Highly Expressed in Head and Neck Squamous Carcinoma (HNSC) and NPC

**Figure 1:**
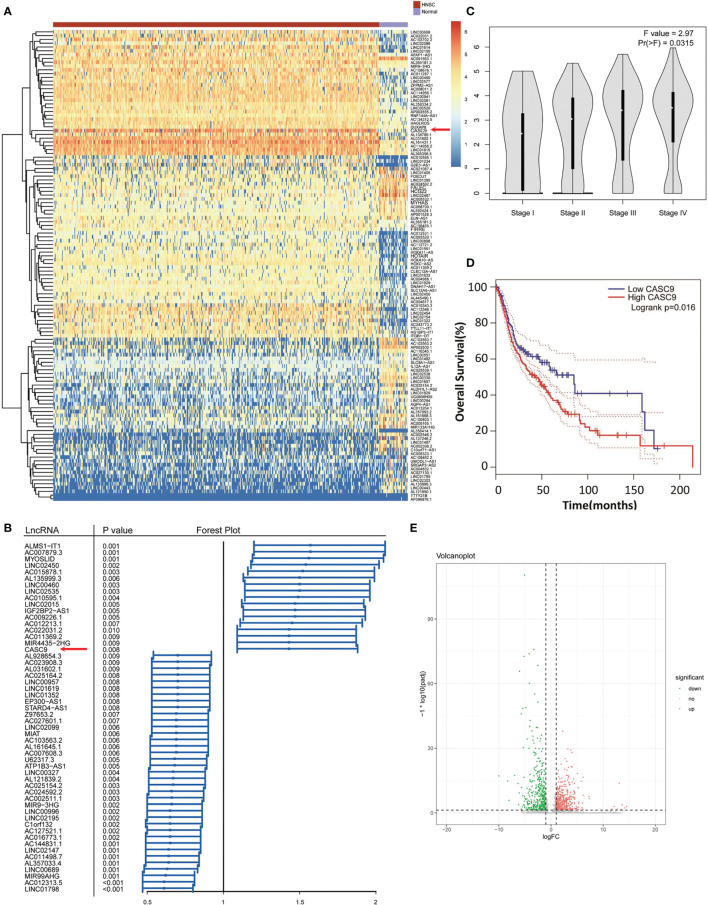
CASC9 were overexpressed and predicted adverse clinical outcomes in HNSC and NPC based on bioinformatics analysis. **(A)** The DElncRNAs in TCGA-HNSC dataset were shown in the thermograph, including CASC9. **(B)** Forest plot of the prognosis-associated DElncRNAs including CASC9(*P <*0.008). **(C)** Higher CASC9 expression was related to advanced TNM staging in HNSC. **(D)** Higher CASC9 expression was related to HNSC patients’ shorter OS. **(E)** Volcano Plot visualized the DElncRNAs in NPC.

To find promising differentially expressed lncRNAs (DElncRNAs) candidates in HNSC, we conducted comprehensive lncRNAs profile analyses using TCGA-HNSC dataset. A total of 889 DElncRNAs were screened, in which the number of upregulated and downregulated lncRNAs was 497 and 392 respectively (**Supplementary Table 3**). As shown in **Figure 1A**, we found CASC9 were significantly up-regulated in HNSC. Based on Cox regression analysis, 54 prognosis-associated DElncRNAs was further selected out (**Figure 1B**). Meanwhile, we found that higher CASC9 expression was positively correlated with advanced TNM stage and shorter overall survival in HNSC (*P*=0.0315 and *P*=0.016, **Figures 1C, D**). To confirm the discovery in HNSC, we identified DElncRNAs in NPC and found that expression of CASC9 was similarly higher in NPC compared with normal tissues based on two independent NPC RNA-seq datasets (**Figure 1E** and **Supplementary Tables 4**, **5**) (19, 20). By reviewing literatures, we discovered that CASC9-related researches is still very limited and the role of CASC9 in tumor progression remains unknown. Therefore we concentrated on the CASC9 to test whether it was possible to function in tumorigenesis or not.

**Figure 2:**
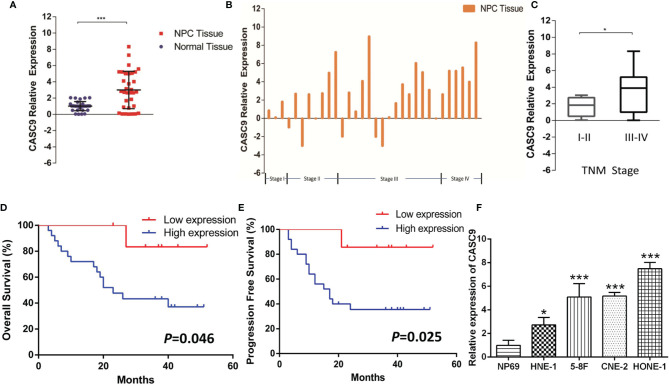
Relative expression of CASC9 in human NPC tissues and cell line. **(A, B)** CASC9 was up-regulated in NPC tissues compared with corresponding adjacent non-tumor tissues (*P* < 0.001). **(C)** CASC9 in NPC tissues of patients in stage III-IV was significantly higher than that of patients in stage I-II (*P* < 0.05). **(D, E)** Kaplan–Meier curve of overall survival and progression free survival of NPC patients with high and low CASC9 expression. **(F)** Expression level of CASC9 in a panel of human NPC cell lines (HNE-1, 5-8F, CNE-2, HONE-1) and normal epidermal cell NP-69. *P < 0.05, ***P < 0.001.

We measured the expression abundance of CASC9 in 32 pairs of NPC and adjacent non-tumor samples as shown in **Figures 2A, B**. As expected, expression of CASC9 was overexpressed in 78.13% NPC tissues (25/32, *P*<0.001). Moreover, we discovered that the expression of CASC9 of patients in stage III-IV is significantly higher than that of patients in stage I-II (*P*<0.05, **Figure 2C**). Kaplan–Meier analysis indicated that NPC patients with higher CASC9 expression were associated with poorer overall survival and shorter progression-free survival (*P*=0.046 and *P*=0.025, **Figures 2D, E**). Similarly, the expression of CASC9 was significantly increased in HNE-1, 5-8F, CNE-2 and HONE-1 cell lines, compared to the human nasopharyngeal epithelial cell line NP69 (**Figure 2F**).

### CASC9 Promotes NPC Cell Growth *In Vitro*

**Figure 3:**
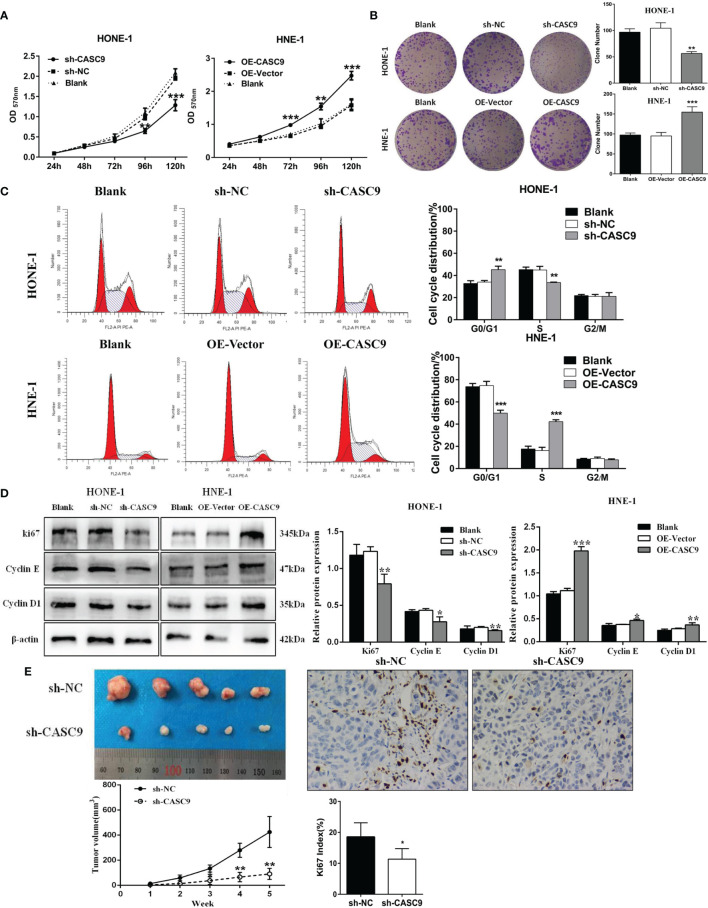
CASC9 promoted NPC cell growth *in vitro* and *in vivo*. **(A, B)** The proliferation changes of NPCs were determined using MTT assay and colony-formation assay. **(C)** Cell cycle was analyzed by flow cytometry. **(D)** Protein levels of Cyclin D1, Cyclin E and Ki67 were measured by western blotting. **(E)** The knockdown of CASC9 inhibited NPC tumor growth *in vivo*. The expression of Ki67 was determined using immunohistochemistry. *P < 0.05, **P < 0.01, ***P < 0.001.

To explore the biological role of CASC9 in NPC progression, knockdown experiment were performed using HONE-1 cells, and overexpression experiments were conducted using the HNE-1 cells. The MTT assay showed that knockdown of CASC9 significantly inhibited the proliferation of HONE-1 cells (*P*<0.001) and overexpression of CASC9 promoted the proliferation of HNE-1 cells (*P*<0.001, **Figure 3A**). Cells transfected with CASC9 shRNA resulted in fewer colonies than sh-NC group (*P*<0.01). Conversely, the number of colonies formed by HNE-1 cells increased obviously after CASC9 overexpression (*P*<0.01, **Figure 3B**). Cell cycle was evaluated by flow cytometry analysis following knockdown or overexpression of CASC9. As shown in **Figure 3C**, CASC9 knockdown arrested the cell cycle at G0/G1 phase in HONE-1 cells, while CASC9 overexpression promoted HNE-1 cell cycle progression. The down-regulation of CASC9 led to a significant decrease in the cell cycle-related protein Cyclin D1, Cyclin E and Ki67 protein abundances (**Figure 3D**). On the contrary, the relative expression level of Ki67, Cyclin D1 and Cyclin E increased in HNE-1 after up-regulation. Collectively, high expression of CASC9 promotes NPC cell growth *in vitro*.

### The Knockdown of CASC9 Inhibits Xenograft Tumor Growth

In order to further explore the effect of CASC9 on the growth of nasopharyngeal carcinoma cells *in vivo*, HONE-1 cells transfected with sh-CASC9 or sh-NC were introduced into nude mice. According to the growth curves, tumor growth in the sh-CASC9 group was evidently suppressed compared with control group from the third week after inoculation (*P*<0.01, **Figure 3E**). All nude mice were sacrificed on the 35th day after inoculation, and the subcutaneous tumors were stripped. As shown in **Figure 3E**, the tumor size of the sh-CASC9 group was significantly smaller than that of the control group. In addition, we found that the deficiency of CASC9 led to a reduction in Ki67 expression. Our findings indicated that knockdown of CASC9 could inhibit xenograft tumor growth.

### CASC9 Promotes the Migration and Invasion of NPC Cells

**Figure 4:**
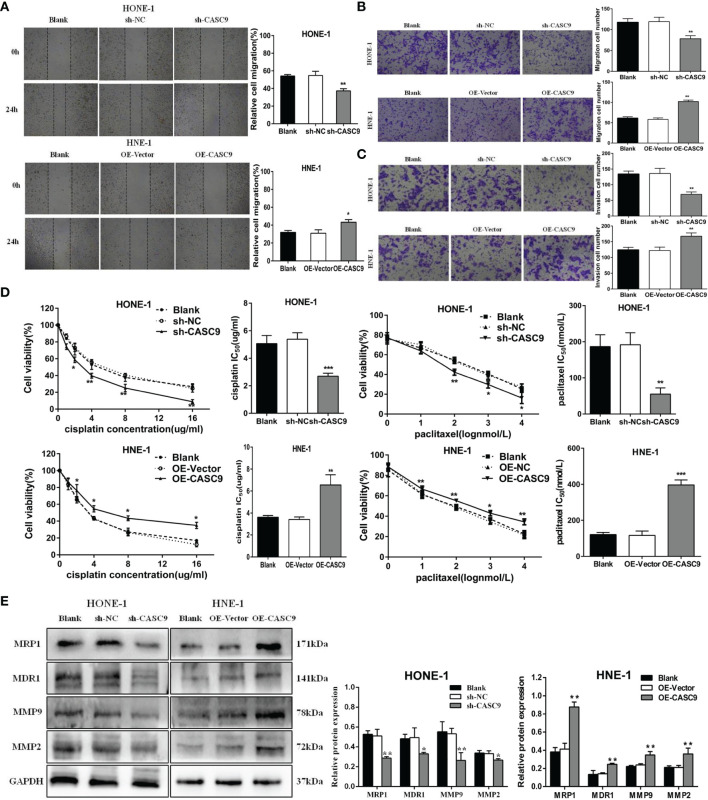
CASC9 facilitated NPC cells migration and invasion and decreased the sensitivity to cisplatin and paclitaxel. **(A, B)** The migratory abilities of NPC cells were determined using wound healing assay and transwell assay. **(C)** The invasion abilities of NPC cells were determined using transwell assay. **(D)** Knockdown of CASC9 expression enhanced the sensitivity of NPC cells to cisplatin and paclitaxel. **(E)** The expression of related proteins were determined using western blotting. *P < 0.05, **P < 0.01, ***P < 0.001.

Cellular migration abilities were detected by wound healing and transwell assay. The results suggested that knockdown of CASC9 expression could significantly inhibit the migration ability of HONE-1 cells, while overexpression of CASC9 remarkably increased migration of HNE-1 cells (**Figures 4A, B**). Invasion analysis showed that HNE-1 cells expression higher level of CASC9 had stronger capability for invasion compared with that of control cells, while HONE-1 cells harboring lower level of CASC9 had a great loss in invasion ability (**Figure 4C**). As shown in **Figure 4E**, the inhibition of CASC9 evidently inhibited the expressions of the MMP9 and MMP2 proteins in the HONE-1 cells. On the contrary, the expression of MMP9 and MMP2 were increased by overexpression of CASC9 in the HNE-1 cells. These results demonstrated that high expression of CSAC9 facilitates migration and invasion of NPC cells.

### Knockdown of CASC9 Expression Enhances the Sensitivity of NPC Cells to Cisplatin and Paclitaxel

To explore the relationship between CASC9 and the chemotherapy sensitivity of NPC cells, we identify the sensitivity of cells to cisplatin and paclitaxel by using MTT assay. Our data revealed that in contrast to homologous control, knockdown of CASC9 strikingly decreased the IC50 values of cisplatin (*P*<0.001) and paclitaxel (*P*<0.01) in HONE-1 cells (**Figure 4D**). Consistent with this, HNE-1 cells with CASC9 overexpression presented higher cell survival rate to cisplatin and paclitaxel. It is also worth noting that the expression of drug resistance-associated proteins MRP1 and MDR1 were significantly decreased in cells treated with sh-CASC9, which is consistent with the results uncovered in MTT analysis (**Figure 4E**).

### miR-497-5p Is A Direct Target of CASC9 in NPC Cells

**Figure 5:**
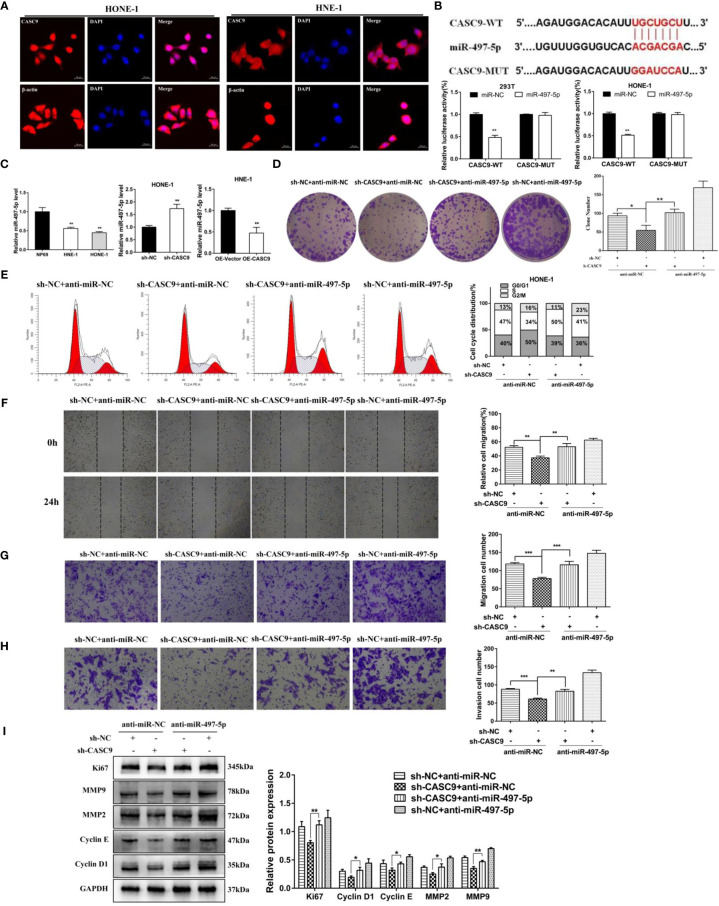
miR-497-5p was a direct target of CASC9 and the abrogation of miR-497-5p reversed the inhibitory effects of the CASC9 knockdown on NPC cells. **(A)** RNA-FISH results revealed that CASC9 was distributed in both nucleus and cytoplasm of NPC cells. **(B)** The binding sites of miR-497-5p and CASC9 were predicted through bioinformatics analysis and confirmed by dual-luciferase reporter gene assay. **(C)** miR-497-5p expression was decreased and negatively associated with CASC9 in NPC cells. **(D, E)** Knockdown of miR-497-5p reversed the effect of silencing CASC9 on cell proliferation and cell cycle. **(F, G)** Knockdown of miR-497-5p reversed the effect of silencing CASC9 on cell migration. **(H)** Knockdown of miR-497-5p reversed the effect of silencing CASC9 on cell invasion. **(I)** The expression of proliferation and invasion-related proteins were determined using western blotting. *P < 0.05, **P < 0.01, ***P < 0.001.

We performed RNA-FISH in both HONE-1 and HNE-1 cells to identify the subcellular localization of CASC9. It revealed that CASC9 was distributed in both nucleus and cytoplasm of NPC cells (**Figure 5A**). Then we found that CASC9 had shared putative binding sites with miR-497-5p. The prediction was confirmed by dual luciferase reporter gene assay. The results indicated that the miR-497-5p transfection reduced the luciferase activity in cells transfected with CASC9-WT (*P*<0.01), but no significant effect was found in cells transfected with CASC9-MUT (**Figure 5B**).

### miR-497-5p Is Low Expressed in NPC Cells and Negatively Regulated by CASC9

We found that the expression of miR-497-5p was abnormally downregulated in NPC cells compared with that in NP69 cells through using qRT-PCR (*P*<0.01, **Figure 5C**). Moreover, **Figure 5C** also showed that knockdown of CASC9 increased miR-497-5p expression in HONE-1 cells, while overexpression of CASC9 would inhibit the expression of miR-497-5p in HNE-1 cells. This results revealed that miR-497-5p was negatively regulated by CASC9.

### Knocking Down miR-497-5p Could Reverse the Effect of Silencing CASC9 on the Proliferation, Migration And Invasion

To further explore the relationship between miR-497-5p and CASC9, we performed miR-497-5p blocking experiments. Our results indicated that the knockdown of miR-497-5p obviously reversed the effect of silencing CASC9 on the proliferation (**Figures 5D, E**), migration (**Figures 5F, G**), invasion (**Figure 5H**). Besides, we detected the expression of proliferation and invasion-related proteins, which suggested that knocking down miR-497-5p weakened the sh-CASC9-mediated the reduction of the expressions of the Ki67, CyclinD1, CyclinE, MMP2, MMP9 proteins in the HONE-1 cells (**Figure 5I**).

### Wnt3a Is Highly Expressed in NPC Cells and Negatively Regulated by miR-497-5p

**Figure 6:**
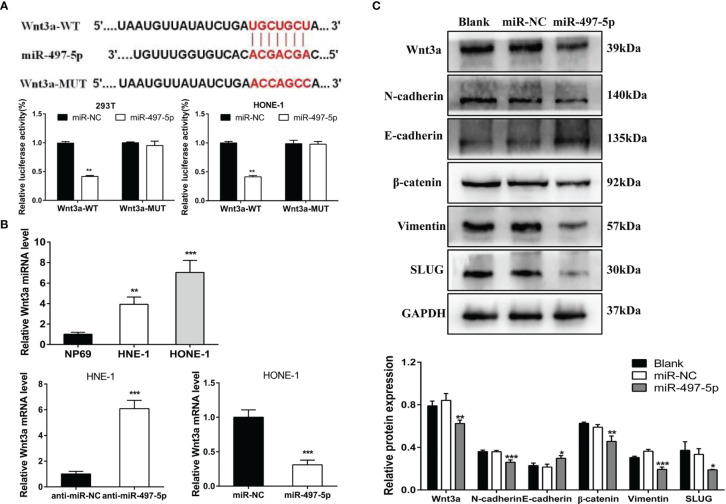
miR-497-5p affected the wnt3a/β-catenin pathway. **(A)** The binding sites of Wnt3a and miR-497-5p were predicted through bioinformatics analysis and confirmed by dual-luciferase reporter gene assay. **(B)** Wnt3a expression was increased and negatively associated with miR-497-5p in NPC cells. **(C)** Overexpression of miR-497-5p led to a significant inhibition of Wnt3a/β-catenin signaling pathway. *P < 0.05, **P < 0.01, ***P < 0.001.

The putative binding sites of the miR-497-5p and Wnt3a were predicted by TargetScan algorithm. Dual luciferase reporter gene assay indicated that the miR-497-5p mimics transfection reduced the luciferase activity in cells transfected with Wnt3a-WT (*P*<0.01), but no significant effect was found in cells transfected with Wnt3a-MUT (**Figure 6A**). Moreover, overexpression miR-497-5p decreased the expression for Wnt3a, which was highly expressed in NPC cells (**Figure 6B**). This results revealed that Wnt3a was negatively regulated by miR-497-5p.

In order to understand whether miR-497-5p affects Wnt3a/β-catenin pathway, we detected the expression of Wnt3a/β-catenin pathway related proteins after miR-497-5p overexpression in nasopharyngeal carcinoma cells by Western blot. The results showed that the excessiveness of miR-497-5p led to a significant decrease in Wnt3a, β-catenin, N-cadherin, Vimentin and SLUG protein expressions and an increase in E-cadherin (**Figure 6C**).

## Discussion

Intensity modulated radiation, chemotherapy and immune checkpoint inhibitors have been successively applied in clinical therapy, however, the prognosis of NPC still remains poor (5, 21, 22). Despite several mechanisms have been reported, most of molecular features underlying the pathogenesis of NPC remain unknown. Increasing evidences are improving the understanding that lncRNAs play crucial roles in the pathogenesis and development of many cancers. Therefore, it is important to elucidate the molecular mechanism of prognosis-related lncRNAs and explore potential therapeutic targets.

In this study, we found that CASC9 was highly expressed in NPC tissues and negatively associated with NPC patients’ survival. Knockdown of CASC9 inhibited the proliferation, migration and invasion of NPC cells, which is in agreement with previous studies (15, 18). Besides, we firstly demonstrated that silencing CASC9 enhances the sensitivity of NPC cells to cisplatin and paclitaxel, which is also consistent with the research in gastric carcinoma (12). However, as an oncogene in tumor progression, the underlying mechanism allows CASC9 participating in NPC progression remains poorly understood. Previous researches showed that CASC9 could act as miRNA sponge to regulate the biological behavior of tumor cells. For instance, CASC9 could sponge miR−758−3p to promote cell proliferation and epithelial−mesenchymal transition in bladder cancer by regulating TGF−β signaling pathway (23). In addition, a report also found that CASC9 could promote non-small cell lung cancer progression *via* miR-335-3p/S100A14 axis (24). Bioinformatics analysis and luciferase reporter assay indicated the binding sites between CASC9 and miR-497-5p in current study.

Some studies have suggested that miR-497-5p could inhibit the proliferation and invasion of triple-negative breast cancer cells by targeting CCNE1, block the cell cycle and promote cell apoptosis (25). In addition, Wang et al. revealed that miR-497-5p is a potent tumor suppressor that inhibits cancer phenotypes in NPC (26). Similarly, we found low expression of miR-497-5p in NPC cells in our research. Notably, miR-497-5p absence could reverse the effect of silencing CASC9 on the proliferation, migration, invasion of NPC cells. These results strongly suggested that miR-497-5p is a direct target of CASC9.

However, the molecular mechanisms that underlie the tumor suppressive role of miR-497-5p remains unknown. In the present study, the putative binding sites of the miR-497-5p and Wnt3a were predicted *via* bioinformatic analysis and verified by luciferase reporter assay. Wnt3a, as one of Wnt family members, plays a critical role in initiation, progression, invasion and metastasis of cancer (27, 28). Relevant studies have found that Wnt3a is significantly highly expressed in gastric cancer (29), prostate cancer (30) and breast tumors (31). In recent studies of osteoarthritis, Wnt3a was confirmed as a direct target of miR-497-5p, and expression of miR-497-5p was negatively correlated with Wnt3a level in cartilage (32). However, the interaction between Wnt3a and miR-497-5p in malignant tumors has not been reported yet. In present study, it was firstly demonstrated that Wnt3a was upregulated in NPC cells and negatively associated with miR-497-5p. Overexpression of miR-497-5p led to a significant inhibition of Wnt3a/β-catenin signaling pathway. Therefore, we provided insight into CASC9 regulating NPC cell proliferation, migration, invasion and chemosensitivity *via* interacting with miR-497-5p/Wnt3a.

In conclusion, the present study revealed that CASC9 was up-regulated in NPC tissues and cells, indicating poor prognosis. CASC9 knockdown inhibited the proliferation, migration and invasion of NPC cells and enhanced the sensitivity of tumor cells to cisplatin and paclitaxel. CASC9 may regulate the malignant biological behavior of nasopharyngeal carcinoma cells by targeting miR-497-5p/Wnt3a/β-catenin signaling pathway. The present study might provide a novel mechanism for progression and pathogenesis of NPC and contribute to the development of an effective molecular target therapy.

## Data Availability Statement

The datasets presented in this study can be found in online repositories. The names of the repository/repositories and accession number(s) can be found in the article/**Supplementary Material**.

## Ethics Statement

The studies involving human participants were reviewed and approved by the Ethics Committee of the Second Affiliated Hospital of Chongqing Medical University. The patients/participants provided their written informed consent to participate in this study. The animal study was reviewed and approved by the Ethics Committee of the Second Affiliated Hospital of Chongqing Medical University.

## Author Contributions

YL designed the study, analyzed data, and wrote the manuscript. WL, QG, and LL analyzed the data and reviewed the paper. YL, WJ, and WL provided ideas, collected data and reviewed the paper. All authors have read and approved the final manuscript.

## Conflict of Interest

The authors declare that the research was conducted in the absence of any commercial or financial relationships that could be construed as a potential conflict of interest.

## Publisher’s Note

All claims expressed in this article are solely those of the authors and do not necessarily represent those of their affiliated organizations, or those of the publisher, the editors and the reviewers. Any product that may be evaluated in this article, or claim that may be made by its manufacturer, is not guaranteed or endorsed by the publisher.
