# Supplementary material for: Long Non-Coding RNA Cancer Susceptibility Candidate 9 Regulates the Malignant Biological Behavior of Nasopharyngeal Carcinoma Cells by Targeting miR-497-5p/Wnt3a/β-catenin Signaling Pathway
Source: Front Oncol. 2022 Mar 28;12:807052. doi: 10.3389/fonc.2022.807052 (PMC8995468; doi:10.3389/fonc.2022.807052)
Supplement: Supplementary file 1 [file Table_1.docx]

Supplementary Table 1 Clinical features for the NPC patients

| Clinical features |  | Stage I (n=3) | Stage II (n=7) | StageIII (n=16) | Stage IV (n=6) |
| --- | --- | --- | --- | --- | --- |
| Age |  |  |  |  |  |
|  | ≤60 | 2（66.7） | 5（71.4） | 12（75.0） | 4（66.7） |
|  | ＞60 | 1（33.3） | 2（28.6） | 4（25.0） | 2（33.3） |
| Gender |  |  |  |  |  |
|  | male | 3（100.0） | 5（71.4） | 13（81.3） | 4（66.7） |
|  | female | 0 | 2（28.6） | 3（18.7） | 2（33.3） |
| Epstein-Barr virus infection |  |  |  |  |  |
|  | Yes | 2（66.7） | 4（57.1） | 15（93.8） | 3（50.0） |
|  | no | 1（33.3） | 3（42.9） | 1（6.3） | 3（50.0） |
| symptom |  |  |  |  |  |
|  | Nasal symptom | 1（33.3） | 2（28.6） | 4（25.0） | 0 |
|  | Ear symptom | 1（33.3） | 4（57.1） | 5（31.2） | 0 |
|  | Neck mass | 0 | 0 | 5（31.2） | 2（33.3） |
|  | Cranial nerve symptom | 0 | 1（14.3） | 1（6.3） | 3（50.0） |
|  | Others | 1（33.3） | 0 | 1（6.3） | 1（16.7） |
| T stages |  |  |  |  |  |
|  | T1 | 3（100.0） | 5（71.4） | 2（12.5） | 0 |
|  | T2 | 0 | 2（28.6） | 5（31.2） | 0 |
|  | T3 | 0 | 0 | 9（56.3） | 2（33.3） |
|  | T4 | 0 | 0 | 0 | 4（66.7） |
| N stages |  |  |  |  |  |
|  | N0 | 3（100.0） | 2（28.6） | 3（18.7） | 2（33.3） |
|  | N1 | 0 | 5（71.4） | 4（25.0） | 4（66.7） |
|  | N2 | 0 | 0 | 9（56.3） | 0 |
|  | N3 | 0 | 0 | 0 | 0 |
| M stages |  |  |  |  |  |
|  | yes | 0 | 0 | 0 | 2（33.3） |
|  | no | 3（100.0） | 7（100.0） | 16（100.0） | 4（66.7） |
|  |  |  |  |  |  |
