# Supplementary material for: Long Non-Coding RNA Cancer Susceptibility Candidate 9 Regulates the Malignant Biological Behavior of Nasopharyngeal Carcinoma Cells by Targeting miR-497-5p/Wnt3a/β-catenin Signaling Pathway
Source: Front Oncol. 2022 Mar 28;12:807052. doi: 10.3389/fonc.2022.807052 (PMC8995468; doi:10.3389/fonc.2022.807052)
Supplement: Supplementary file 2 [file Table_2.docx]

| CASC9-F | TTGGTCAGCCACATTCATGGT |
| --- | --- |
| CASC9-R | AGTGCCAATGACTCTCCAGC |
| GAPDH-F | CAGCGACACCCACTCCTC |
| GAPDH-R | TGAGGTCCACCACCCTGT |
